# Dysregulation of miR-15a-5p, miR-497a-5p and miR-511-5p Is Associated with Modulation of BDNF and FKBP5 in Brain Areas of PTSD-Related Susceptible and Resilient Mice

**DOI:** 10.3390/ijms22105157

**Published:** 2021-05-13

**Authors:** Oriana Maria Maurel, Sebastiano Alfio Torrisi, Cristina Barbagallo, Michele Purrello, Salvatore Salomone, Filippo Drago, Marco Ragusa, Gian Marco Leggio

**Affiliations:** 1 Section of Pharmacology, Department of Biomedical and Biotechnological Sciences, University of Catania, 95123 Catania, Italy; orianamaurel@hotmail.it (O.M.M.); sebastiano.torrisi@unict.it (S.A.T.); salomone@unict.it (S.S.); f.drago@unict.it (F.D.); 2 Section of Biology and Genetics, Department of Biomedical and Biotechnological Sciences, University of Catania, 95123 Catania, Italy; cbarbagallo@unict.it (C.B.); purrello@unict.it (M.P.)

**Keywords:** AIS model, miRNAs, PTSD, resilience, stress, susceptibility

## Abstract

Post-traumatic stress disorder (PTSD) is a neuropsychiatric disorder occurring in susceptible individuals following a traumatic event. Understanding the mechanisms subserving trauma susceptibility/resilience is essential to develop new effective treatments. Increasing evidence suggests that non-coding RNAs, such as microRNAs (miRNAs), may play a prominent role in mediating trauma susceptibility/resilience. In this study, we evaluated the transcriptional expression of two key PTSD-related genes (FKBP5 and BDNF) and the relative targeting miRNAs (miR-15a-5p, miR-497a-5p, miR-511-5p, let-7d-5p) in brain areas of PTSD-related susceptible and resilient mice identified through our recently developed mouse model of PTSD (arousal-based individual screening (AIS) model). We observed lower transcript levels of miR-15a-5p, miR-497a-5p, and miR-511a-5p in the hippocampus and hypothalamus of susceptible mice compared to resilient mice, suggesting that the expression of these miRNAs could discriminate the two different phenotypes of stress-exposed mice. These miRNA variations could contribute, individually or synergically, to the inversely correlated transcript levels of FKBP5 and BDNF. Conversely, in the medial prefrontal cortex, downregulation of miR-15a-5p, miR-511-5p, and let-7d-5p was observed both in susceptible and resilient mice, and not accompanied by changes in their mRNA targets. Furthermore, miRNA expression in the different brain areas correlated to stress-induced behavioral scores (arousal score, avoidance-like score, social memory score and PTSD-like score), suggesting a linear connection between miRNA-based epigenetic modulation and stress-induced phenotypes. Pathway analysis of a miRNA network showed a statistically significant enrichment of molecular processes related to PTSD and stress. In conclusion, our results indicate that PTSD susceptibility/resilience might be shaped by brain-area-dependent modulation of miRNAs targeting FKBP5, BDNF, and other stress-related genes.

## 1. Introduction

Intense stressful events evoke a plethora of biological responses, including neurochemical cascades, altering the functioning of the sympathetic–adrenal–medullary (SAM) system, the hypothalamic–pituitary–adrenal (HPA) axis, and the immune system. These biological changes can modify the neuronal connectivity, signaling, and remodeling and are associated with the exacerbation or development of neuropsychiatric diseases, such as depression and post-traumatic stress disorder (PTSD) [1,2,3]. Cellular and molecular studies in PTSD and stress-related disorders showed the involvement of a distinct sets of HPA-axis-related genes [4,5,6]. In this context, the roles of FKBP5 (FK506 binding protein 5) and BDNF (brain-derived neurotropic factor) have been reported to be strongly associated to PTSD etiopathogenesis [7,8,9,10]. FKBP5 belongs to the immunophilin protein family and is a co-chaperone component of GR (glucocorticoid receptor) heterocomplex, playing a role in GR regulation through an ultra-short negative feedback loop activity [11]. Thus, glucocorticoids (GCs) increase FKBP5 expression, which in turn decreases GR affinity for GCs [12]. FKBP5 dysregulation has been associated with a maladaptive stress response [13,14] and its increased expression may be related to HPA axis hyperactivity in humans [15]. Previous studies have documented that FKBP5 knock-out mice exhibited a more resilient phenotype, characterized by lower corticosterone levels, a decreased anxiety-like behavior, better behavioral responses following a social defeat stress, tail suspension test, and forced swim test, and enhanced cognitive flexibility in the radial arm water maze [13,16,17,18]. Moreover, FKBP5 expression in infralimbic medial prefrontal cortex (mPFC) (but not in the central prelimbic cortex) increased after fear conditioning in rats and remained elevated even after extinction, supporting its active role in maladaptive stress response [19]. Consistent with these results, Young et al. found increased levels of FKBP5 in the medial orbitofrontal cortex from postmortem brain tissues of PTSD patients, which positively correlated with dendritic spine density [20].

On the other hand, BDNF, the most studied neurotrophin, is involved in neuroprotection, neurogenesis, neurodevelopment, and synaptic plasticity processes required for long-term learning and memory [7,21,22,23,24]. Notwithstanding evidence showing that acute or chronic stress decreased BDNF expression, which could lead to the loss of synaptic function and increased vulnerability to insults [25,26], other studies reported BDNF overexpression resulting in memory impairments in the forebrain of mice [27], as well as in the hippocampus, hypothalamus, and pituitary gland in chronically stressed rats [28]. A progressive BDNF decrease was also demonstrated at different time points after acute social defeat until 24 h after stress, with levels returning to baseline by day 5 post-stress [29]. Taken together, these results suggest a complex relationship between the intensity and duration of stress on BDNF levels, which would depend significantly also on the type of stress and the brain region being studied. The conflicting effects of stressors on BDNF expression and its related phenotypes show the complexity of stress-related behaviors, and could be the results of different BDNF isoforms with dissimilar functions in various brain areas and differential epigenetic and post-transcriptional regulation, depending on the type of neuron and stimulation [30,31,32,33,34]. FKBP5 and BDNF variations are the result of the complex interactions between environment and gene functions through epigenetic modifications such as DNA methylation, histone modifications, and modulation of non-coding RNAs (ncRNAs) [35,36,37], which play a critical role in adaptive and maladaptive processes [38]. Among ncRNAs, microRNAs (miRNAs) seem to play a pivotal role in neural development and function, as well as in the response to stressors [39,40]. The perturbation of cellular homeostasis may interfere with miRNA biogenesis, leading to deregulation of miRNA-controlled pathways and, accordingly, affecting cell susceptibility to stress [41,42]. The role of miRNA-based epigenetic mechanisms may also play a significant role in the onset and the progression of PTSD [43,44], providing new chances to understand its molecular bases. Interestingly, several recent animal and human studies have shown that miRNAs are present in sperm and may be involved in non-Mendelian inheritance of stress behavioral phenotypes [45,46]. Several animal studies reported that specific miRNAs in particular brain regions are involved in various stress-related behaviors, including fear memory consolidation, contextual fear memory, and state-dependent fear [47,48,49,50,51], suggesting that stress-induced alterations of specific miRNAs are not only temporally, but also spatially defined. Since not everyone who experiences intense acute stress develops PTSD, miRNAs have received increasing attention for their potential contribution to susceptibility and resilience to stress. Furthermore, the potential application of miRNAs as biomarkers of vulnerability/resilience to stress is becoming a focus of particular interest, but only few studies have focused on this issue to date [52,53].

In the present study, we evaluated the expression of a set of miRNAs targeting FKBP5 and BDNF, two key PTSD-related genes, in a new PTSD translational animal model [arousal-based individual screening (AIS)] based on post-trauma changes in startle reactivity, following a single, long, and severe traumatic procedure (24 h restraint stress) [54]. We provide evidence that a single traumatic event induced brain-area-specific miRNA alterations, differentially associated with susceptible and resilient phenotypes, and related to behavioral scores.

## 2. Results

### 2.1. Selection of miRNAs

BDNF and FKBP5 were used as targets to computationally identify predicted and validated miRNAs, both in human and mouse. By this approach, we found 239 miRNAs targeting BDNF and/or FKBP5 both in human or mouse. We further filtered this list by a manually curated search in biomedical literature to identify potential associations between these miRNAs and stress-related disorders. Finally, we focused our experimental analyses on four miRNAs that targeted both BDNF and FKBP5: miR-15a-5p, miR-497a-5p, miR-511-5p, and let-7d-5p.

### 2.2. Expression of miR-15a-5p, let-7d-5p, miR-497a-5p, miR-511-5p, and Their Potential Targets, BDNF and FKBP5

#### 2.2.1. Hippocampus

**Figure 1:**
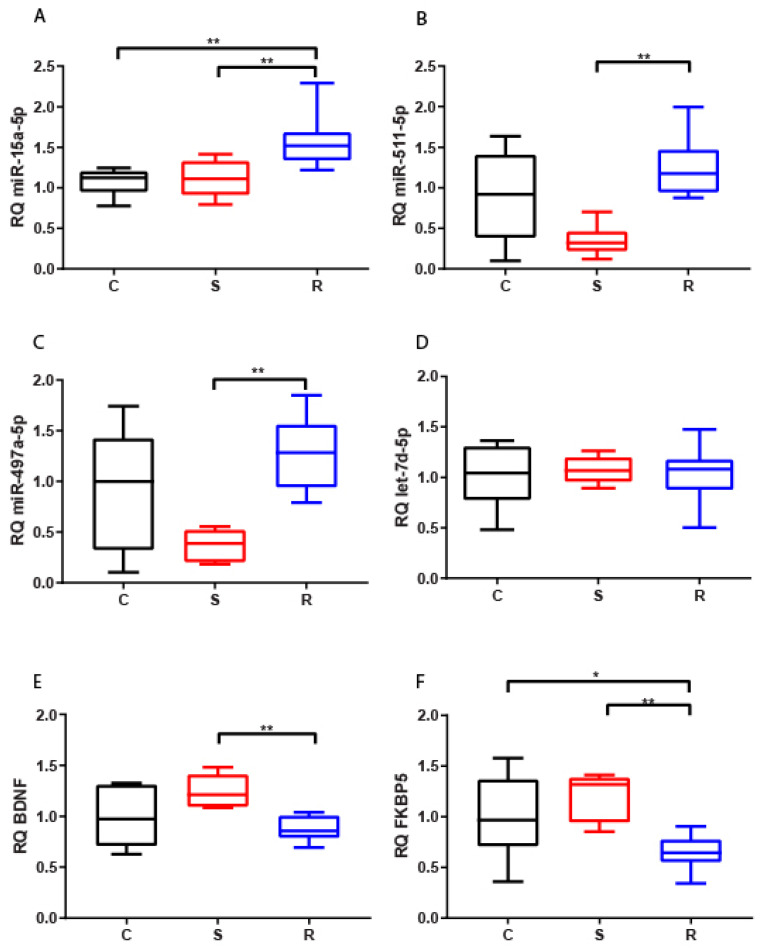
Expression of miR-15a-5p (**A**), let-7d-5p (**B**), miR-497a-5p (**C**), miR-511-5p (**D**), BDNF (**E**), and FKBP5 (**F**) in hippocampi of control (C = 12), susceptible (S = 7), and resilient (R = 11) mice. * *p*-value < 0.05, ** *p*-value < 0.01.

The expression of miR-15a-5p increased in the hippocampus of resilient mice compared to susceptible and control mice (Figure 1A). In the same area, both miR-511-5p and miR-497a-5p showed reduced levels in susceptible animals with respect to resilient mice (Figure 1B,C). These variations taken together suggest that the expression of these three miRNAs could discriminate the two different phenotypes of stressed mice. In contrast, no significant difference was found for hippocampal expression of let-7d-5p (Figure 1D). We observed a statistically significant reduction for both analyzed potential targets of miRNAs, BDNF and FKBP5, in resilient mice compared to susceptible mice, but also in resilient mice compared to control for FKBP5 (Figure 1E,F).

FKBP5 hippocampal expression showed a significant negative correlation with miR-15a-5p, miR-511-5p, and miR-497a-5p, as expected by functional miRNA:mRNA relationships. In contrast, we found no correlation between expression of BDNF and miRNAs (Table 1).

#### 2.2.2. Medial Prefrontal Cortex

**Figure 2:**
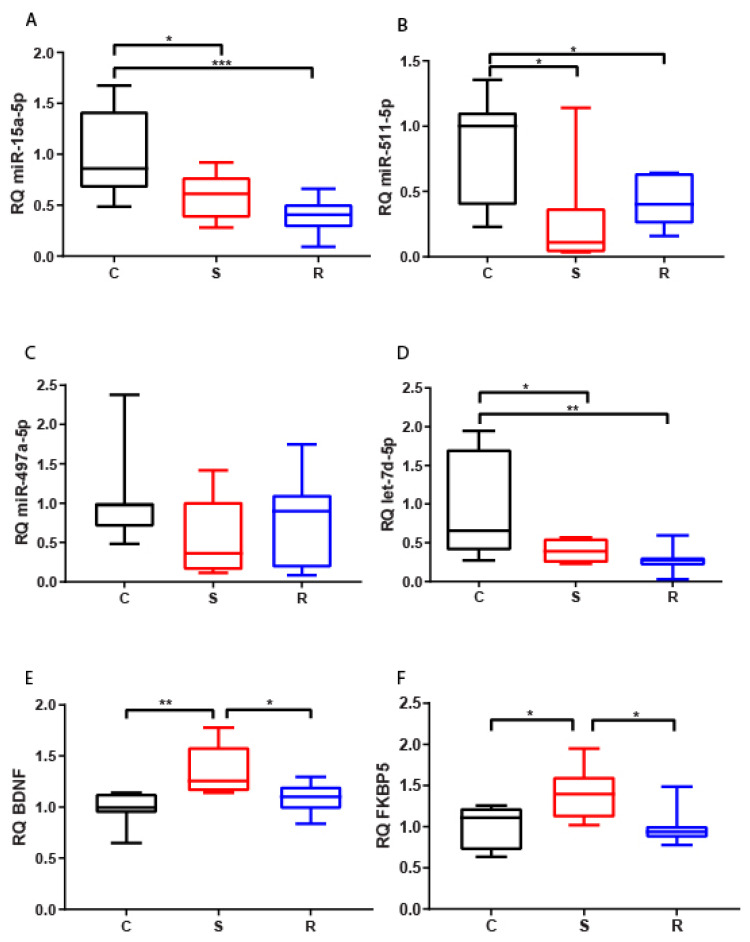
Expression of miR-15a-5p (**A**), let-7d-5p (**B**), miR-497a-5p (**C**), miR-511-5p (**D**), BDNF (**E**), and FKBP5 (**F**) in mPFCs of control (C = 12), susceptible (S = 7), and resilient (R = 11) mice. * *p*-value < 0.05, ** *p*-value < 0.01, *** *p*-value < 0.001.

We found a strong reduction of miR-15a-5p, miR-511-5p, and let-7d-5p levels in mPFC in both subpopulations of stressed mice compared to controls, except for miR-497a-5p, whose expression alteration was not statistically significant (Figure 2A–D). These findings suggest that low levels of these miRNAs in this specific brain area are associated with a general reaction to traumatic stress, but not with the discrimination of resilient and susceptible phenotypes. On the other hand, both FKBP5 and BDNF increased their expression in susceptible mice with respect to control and resilient mice (Figure 2E,F).

The inconsistent expression variations between miRNAs and their potential mRNA targets was also demonstrated by calculated correlations. Indeed, we found no significant linear relationship between miRNAs and targets (Table 1).

#### 2.2.3. Hypothalamus

**Figure 3:**
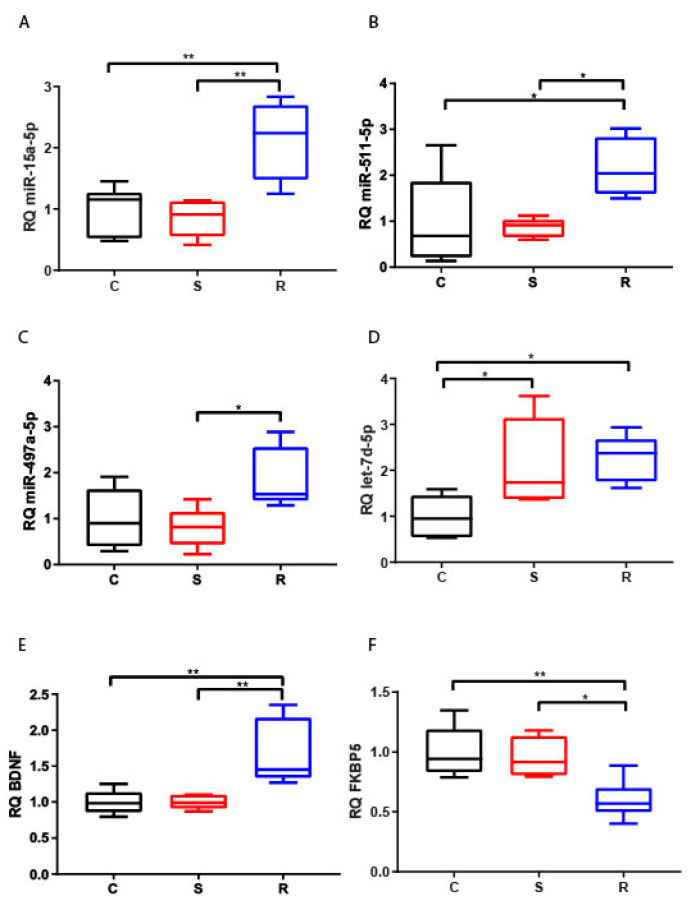
Expression of miR-15a-5p (**A**), let-7d-5p (**B**), miR-497a-5p (**C**), miR-511-5p (**D**), BDNF (**E**), and FKBP5 (**F**) in hypothalami of control (C = 6), susceptible (S = 5), and resilient (R = 5) mice. * *p*-value < 0.05, ** *p*-value < 0.01.

The hypothalamic expression of miR-15a-5p, miR-511-5p, and miR-497a-5p (Figure 3A–C) significantly increased in resilient mice compared to susceptible mice, and also in comparison with control mice for miR-15a-5p and miR-511-5p. However, both stressed groups showed an evident upregulation of let-7d-5p compared to control animals (Figure 3D). We observed a decrease of FKBP5 mRNA expression in resilient mice compared to both susceptible and control mice, whilst an upregulation of BDNF was evident in the same comparisons (Figure 3E,F).

In this brain area, miRNAs showed an opposite trend of deregulation with respect to FKBP5, but not to BDNF, as supported by the significant negative correlations between FKBP5 and miR-15a-5p, miR-511-5p and miR-497a-5p (Table 1).

#### 2.2.4. Evaluation of Synergic Effect of miRNAs on FKBP5 and BDNF Expression

We also investigated whether the four PTSD-related miRNAs may have a synergic effect on regulating FKBP5 and BDNF expression, being both targeted by all the analyzed miRNAs. Different linear regression models were computed, each one including all four miRNAs, three miRNAs, and two miRNAs, considering all possible combinations. For each model, we analyzed all the combinations of miRNAs, considering only those miRNAs showing a negative linear correlation with the target, as previously reported in Table 1. Table 2 lists the models showing the highest R-value for each target in each brain area.

According to R^2^ values, which represent the proportion of the variance for the dependent variable that may be explained by the independent variables, this analysis suggests that part of FKBP5 or BDNF expression may be explained by the synergic regulation performed by the PTSD-related miRNAs. In particular, as regards FKBP5, more than 41% of its expression in the hippocampus and 51% in the hypothalamus may be explained by variation of miRNAs, with the best result obtained by the combination of all four miRNAs

#### 2.2.5. Potential Co-Regulation of PTSD-Related miRNAs

Since the analyzed miRNAs frequently showed similar variation trends in the same brain area in stressed mice, we investigated whether their expressions were statistically correlated with each other in HIP, HT, and, mPFC (Table 3).

**Figure 4:**
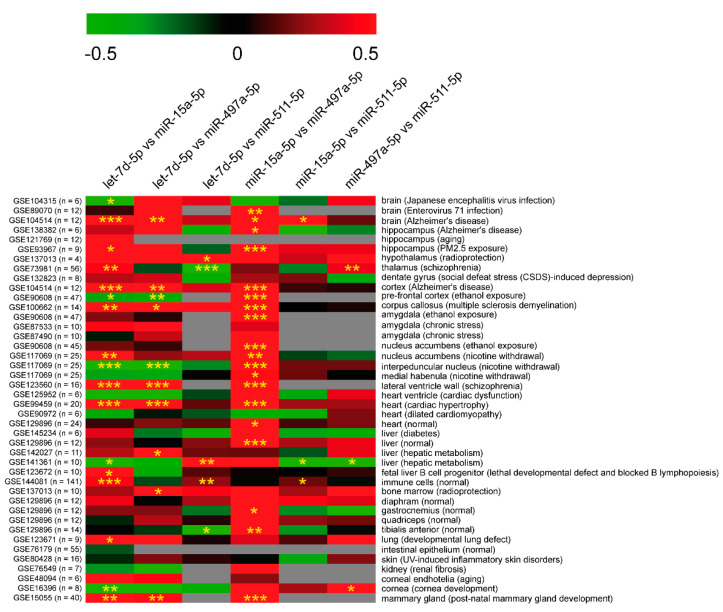
Correlation matrix of miRNA expression from GEO data in *Mus musculus*. The correlation coefficient is indicated by a color gradient from green (negative correlation) to red (positive correlation), as shown in the colored bar. GEO IDs and number of samples (between brackets) are reported on the left of the matrix. Grey is used for datasets where correlation for the specific pair was impossible to calculate (expression data for at least one of the miRNAs were not available). Tissues and diseases (between brackets) are reported on the left of the matrix. Statistically significant *p*-values are indicated by asterisks: * *p*-value < 0.05, ** *p*-value < 0.01, *** *p*-value < 0.001.

**Figure 5:**
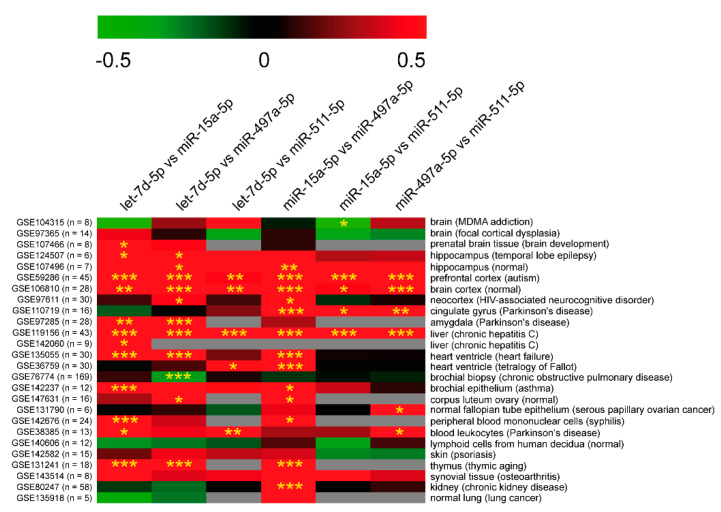
Correlation matrix of miRNA expression from GEO data in *Homo sapiens*. The correlation coefficient is indicated by a color gradient from green (negative correlation) to red (positive correlation), as shown in the colored bar. GEO IDs and number of samples (between brackets) are reported on the left of the matrix. Grey is used for datasets where correlation for the specific pair was impossible to calculate (expression data for at least one of the miRNAs were not available). Tissues and diseases (between brackets) are reported on the left of the matrix. Statistically significant *p*-values are indicated by asterisks: * *p*-value < 0.05, ** *p*-value < 0.01, *** *p*-value < 0.001.

In HIP, we found a statistically significant positive correlation between the following miRNAs: miR-15a-5p and let-7d-5p, miR-15a-5p and miR-511-5p, miR-511-5p and miR-497a-5p, and miR-511-5p and let-7d-5p. In contrast, no significant correlation was observed between miR-497a-5p and, respectively, miR-15a-5p and let-7d-5p. In HT, we found a miRNA:miRNA correlation pattern consistent with the one observed in HIP. In fact, miR-15a-5p was positively correlated with miR-511-5p and let-7d-5p, while miR-511-5p showed the same significant relationship with miR-497a-5p and let-7d-5p. No significant linear relationship was observed between miR-497a-5p and miR-15a-5p or let-7d-5p. In mPFC, we found significant positive correlations for the pairs miR-497a-5p:miR-15a-5p and miR-497a-5p:let-7d-5p, and a strong linear relationship between miR-15a-5p and let-7d-5p, as previously observed in HIP. As we found several positive correlations of expression among all miRNAs in all analyzed areas, we corroborated this observation by screening public miRNA expression databases. We retrieved miRNA expression data from different tissues, including brain areas, from mouse and human datasets deposited in the GEO database. Pearson’s correlation coefficients confirmed that expressions of let-7d-5p, miR-15a-5p, miR-497a-5p, and miR-511-5p were frequently co-linear in many mouse and human tissues, especially in the brain, in agreement with results obtained in our PTSD-like model (Figure 4 and Figure 5).

Based on these data, we speculated on the possibility that these miRNAs could share some transcriptional and post-transcriptional regulators. For this purpose, we compared the transcription factors (TFs) binding miRNA promoters and the long non-coding RNAs (lncRNAs) predicted to decoy (bind and sequester) these miRNAs in mouse. This comparative analysis showed that regulatory regions of miR-15a and miR-497a are potentially bound by 27 common TFs, as expected because they belong to the same miRNA family. The genomic loci of all the analyzed miRNAs are characterized by promoter regions that immunoprecipitate with the TFs Cebpa/Cebpb and Spi1 (Table 4).

Moreover, miR-15a-5p, let-7d-5p, miR-511-5p, and miR-497a-5p share RNA-binding sites for several lncRNAs. More specifically, Kcnq1ot1 (KCNQ1 overlapping transcript 1) and Gm4117 are predicted to sequester all the PTSD-related miRNAs (Table 5).

#### 2.2.6. Correlation Analysis Between RNA Expression and Behavioral Scores

Since we observed a dysregulated expression of mRNAs and miRNAs in different brain areas in association with susceptible and resilient phenotypes, we evaluated whether RNA alterations were linearly related to the composite scores of behavioral tests in susceptible and resilient mice, including arousal score, avoidance-like score, social memory score, and PTSD-like score (Table 6).

In HIP, we found a statistically significant inverse correlation between arousal score and expression levels of miR-15a-5p and miR-511-5p and, in contrast, a congruent positive association between the same score and BDNF and FKBP5. These data suggest that the arousal level of mice segregated through the AIS model could be controlled by the hippocampal levels of BDNF and FKBP5, and their targeting miRNAs miR-15a-5p and miR-511-5p. On the other hand, we found no significant correlation between scores and expression of miRNAs and mRNAs in mPFC. These data were consistent with the observation that miRNA dysregulation in mPFC was associated with traumatic stress independently of susceptibility or resilience. Finally, we found a strong anti-correlation between avoidance-like score and hypothalamic expression of both miR-511-5p and BDNF. The expression of miR-497a-5p in the hypothalamus was negatively correlated with arousal, avoidance-like score, and PTSD-like scores. Since we investigated the expression of multiple miRNAs and mRNAs, we evaluated whether their levels could be synergically associated with behavior. Linear regression analysis was performed considering all miRNAs and mRNAs together, or only miRNAs and only mRNAs. Similar to previous linear regression analyses, R^2^ evaluation showed that miRNAs alone account for 96%, 78%, and 83% of arousal, social, and PTSD-like score, respectively, in the hypothalamus. Moreover, in the hippocampus the combination of miRNAs and mRNAs may affect the arousal score (51%), while mRNAs alone may slightly contribute to it (27%) (Table 7).

#### 2.2.7. Dysregulation of miRNAs Affects PTSD-Related Pathways

**Figure 6:**
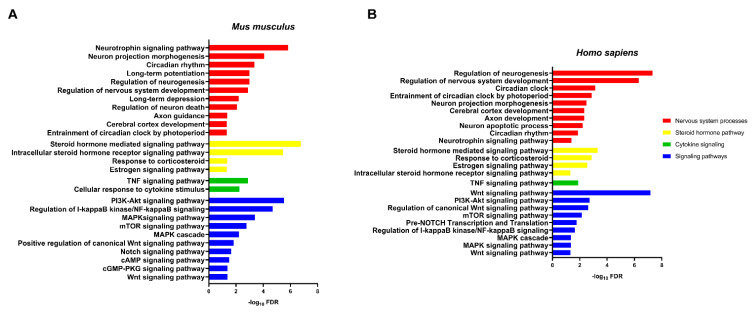
Pathway enrichment analysis in *Mus Musculus* (**A**) and *Homo Sapiens* (**B**). Over-represented biological functions of miR-15a-5p, miR-511-5p, miR-497a-5p, and let-7d-5p were computed by analyzing their validated and predicted targets through the DAVID tool. The over represented pathways are reported on the left of the histograms. Statistical significance is reported as −log_10_ of FDR of Fisher’s exact test.

To further characterize the biological meaning of dysregulation of miR-15a-5p, miR-511-5p, miR-497a-5p, and let-7d-5p, gene ontology and pathway enrichment analyses were performed. The network of genes targeted by the investigated miRNAs is involved in several pathways associated with PTSD both in mice and humans. Indeed, we retrieved several terms related to nervous system processes (such as neurotrophin signaling pathway), response to corticosteroid, cytokine signaling, and several signaling pathways previously associated with PTSD (such as MAPK, Wnt, mTOR, Notch signaling pathway) (Figure 6).

## 3. Discussion

A genetic background is not sufficient alone to properly explain individual differences in sensitivity to stress, given that individual biological diversities are also found within genetically homogeneous populations, such as inbred mice [55]. Life experiences could interact with genetic background to produce long-lasting alterations in coping abilities later in life [56]. For this reason, the development of behavioral and molecular profiling, discriminating susceptible and resilient mice to identify pro-adaptive or maladaptive mechanisms, is fundamental. In the present study, we investigated the expression of two key PTSD-genes, FKBP5 and BDNF, and their targeting miRNAs in a long-term PTSD mouse model, in which a single severe restraint of long duration is able to trigger persistent PTSD-like phenotypes in susceptible mice [54]. Animal studies have shown that dysregulation of miRNA levels in different brain regions can be associated with behavioral alterations [57]. The MiR-15/107 family is highly expressed in brain tissues and comprises multiple highly conserved miRNA members, including miR-497a-5p [58]. Our data would suggest that the consistent variations of miR-15a-5p, miR-497a-5p, and miR-511-5p and their targets in the hippocampus and hypothalamus could potentially promote the resiliency processes by contributing to neuroadaptation [59]. On the other hand, miR-15a-5p, miR-511-5p, and let-7d-5p levels were reduced in the mPFC of all stressed animals, suggesting that these miRNAs are not specifically involved in vulnerability and resiliency phenomena in this brain area, but rather in biological events related to the stress. Moreover, their expression was unrelated to BDNF and FKBP5, suggesting that mRNA modulation is also controlled by other molecular factors in this brain area. Although our molecular and computational data derived from a new mouse model of PTSD, accumulating evidence suggests the involvement of these miRNAs in stress-related disorders, as well as in neurological diseases, thus supporting the results of this study [60]. In this context, Volk et al. showed reduced levels of miR-15a in the amygdala of mice exposed to chronic stress exhibiting an increase of anxiety-like behavior [61]. Others groups confirmed the involvement of miR-511 in the risk of stress-related disorders, including major depression and PTSD, even if its role in susceptibility is still unclear [62,63]. Depressed patients had reduced miR-511 expression in the prefrontal cortex, as well as rats tested in the chronic unpredictable mild stress (CUMS) paradigm [62,63,64]. Some studies showed that miR-497 levels were low in the prefrontal cortex of depressed suicide patients, while significantly increased levels were observed in schizophrenic subjects, suggesting an involvement of miR-497 in psychiatric disorders [65,66,67]. Unlike the other analyzed miRNAs, levels of let-7d-5p did not change in the hippocampus, increased in the hypothalamus and decreased in mPFC in stressed mice. These results may suggest an involvement of let-7d-5p in stress-related disorders, but not in the discrimination of vulnerable and resilient phenotypes. Growing evidence indicates that let-7d may play a role in the neurobiology of psychiatric disorders [68]. Let-7d is largely expressed in the brain and is involved in modulating learning and memory [69,70]; however, little is known about its role in stress-related disorders. Interestingly, its upregulation was reported in the nucleus accumbens in a cocaine-conditioned place preference rat model and, in its circulating form, in animals that had undergone chronic social defeat [52,71]. Specific temporal and spatial expression of miRNAs in the brain is known, but the role of the area specificity in gene regulatory networks has not been satisfactorily addressed yet [70]. Our data, together with those previously published in other models, strongly suggest an involvement of these miRNAs in stress-related disorders; in fact, their activity seems to be brain-area-specific and partially associated with the expression of their potential targets. This last observation would suggest that these miRNAs could also target different mRNAs other than BDNF and FKBP5, as well as that these mRNAs could be regulated by diverse miRNAs or other regulators. Moreover, these PTSD-related miRNAs showed several positive correlations of expression among them in the different brain areas, both in our model and in other human and mouse neuronal and non-neuronal tissues, suggesting that their co-regulation may be due to a common transcriptional or epigenetic control. Indeed, the comparative analysis of miRNA promoters by screening of ENCODE chip-seq experiments showed a potential common regulation by transcription factors Cebpa/Cebpb (CCAAT enhancer binding protein alpha/beta) and Spi1 (transcription factor PU.1) at least for miR-497a-5p and miR-511-5p. Cebpa/b transcriptionally control dozens of genes involved in neuro-differentiation and neurodevelopment in rat hippocampi, and are essential mediators of BDNF/Trk signaling in cortical neurons [72,73]. Although the neuronal role of Spi1 is unknown, its function is critical for viability of brain microglia and its alteration was reported in patients affected by Alzheimer’s disease and major psychiatric disorders [74,75,76]. On the other hand, identification of lncRNAs predicted to bind miR-15a-5p, miR-497a-5p, miR-511-5p, and let-7d-5p showed several common lncRNAs potentially sponging them, thus impairing their function. Among these lncRNAs, Kcnq1ot1 was reported to induce apoptosis or autophagy in neurons after different types of injuries by sequestering different miRNA species [77,78,79]. All these findings would suggest that these miRNAs are part of a complex transcriptional and post-transcriptional machinery that controls fundamental processes in neurons and, consequently, their dysregulation would contribute to molecular cascades induced by neuropsychological stress. Moreover, enrichment analysis of miR-15a-5p, miR-497a-5p, miR-511-5p, and let-7d-5p network showed that such miRNA circuits could affect multiple cellular and molecular processes associated with PTSD pathogenesis [47,80,81,82,83,84,85,86,87,88,89]. Based on these considerations, the inverse correlation between arousal score and hippocampal expression of miR-15a-5p and miR-511-5p is not surprising, as it suggests the existence of a linear relationship between the behavioral arousal score of mice and miRNA levels in the brain that, in turn, coherently influences BDNF and FKBP5 transcripts. Moreover, avoidance-like score in the hypothalamus is negatively correlated with both BDNF and miR-511-5p, hinting that the proportional effects of BDNF on neuronal circuits regulating avoidance-like behaviors are uncoupled by miR-511-5p expression. In the same brain area, miR-497a-5p expression was inversely related to the behavioral arousal, social memory, and PTSD-like scores, suggesting its functional role in PTSD-like phenotypes. Linear regression models showed a synergic effect of the expression of all miRNAs on modulation of behavioral scores in the hypothalamus. This observation, consistent with complex system biology, further indicates that complex phenotypes, such as specific psychiatric behaviors, may be a combined result of expression modulation of different miRNAs, rather than the effect of an individual miRNA [90]. To date, there are only few pre-clinical models based on 24 h restraint stress [91]. Although environmental factors play a predominant role in the individual response to stress, rodent genetic background also modulates stress susceptibility and resilience. Therefore, rodent strain, sex, and biological differences between rats and mice should be considered across experiments to increase reproducibility. For instance, C57Bl/6 strain is reported to be more resilient to stress compared to other strains [92]. There are no comparative studies favoring any acute stress protocol and a single rodent model is not perfectly able to capture human individual complexity [93]. Given that both rats and mice have an important role in the study of neuropsychiatric disorders, it is not surprising that differences in behavior were found, as well as in their molecular biology.

Finally, we believe that the AIS model provides an attractive tool to study the specific role of miRNA-regulated pathways in PTSD susceptibility and resilience. MiRNAs represent a novel class of regulators to molecularly target by using RNA-based therapy. Moreover, even if it is far from our original purpose, we speculate that the miRNAs we found dysregulated could be investigated in body fluids as markers reflecting HPA axis activity and, accordingly, the presence of PTSD phenotype.

## 4. Materials and Methods

### 4.1. Animals

Forty-six male C57BL6/J mice (8 weeks, weight 28 ± 2 gr.) were purchased from Charles River Laboratories (Milano, Italy). Mice were group-housed (3–5 per cage) under controlled conditions (12 h light/dark cycle, 2 V2 ± 2 °C, 55 ± 5% humidity, food and water ad libitum) and weighed once a week until the end of each experiment. All experiments were carried out according to EU Directive 2010/63/EU, the Institutional Animal Care and Use Committees of Catania and the Italian Ministry of Health (authorization n.110/ 2019 PR).

### 4.2. Arousal-Based Individual Screening (AIS), ASR Sessions, Z-Normalization Score, and Behavioral Paradigms

Details regarding the AIS model are described in our previous publication [54]. Briefly, the day before the stress procedure (24 h restraint stress), a pre-trauma acoustic startle reactivity (ASR) session was carried out to measure baseline ASR. The day after, mice were gently put into polyethylene Falcon 50 mL centrifuge tubes and exposed to 24 h of restraint from 3:00 p.m. (3 h before the beginning of the dark phase) to 3:00 p.m. of the next day, without access to food and water. Tubes containing mice were randomly placed in conventional cages, while control mice remained in their home cages in a different room. After 24 h of restraint, mice were suddenly put back in their starting home cages, with free access to food and water. Two other ASR sessions, 14 (ASR 1) and 28 days (ASR 2) after the stress, were given to control and stressed mice in order to assess changes of arousal according to their post-stress ASR changes (% of ASR baseline). To segregate stressed mice into susceptible and resilient subgroups, we created an arousal score by applying a mathematical approach (Z-normalization) to ASR changes. After the segregation, susceptible and resilient mice were housed in the same starting cages. Stressed mice that exhibited an arousal score ≥ 1 were classified as susceptible, while stressed mice with an arousal score < 1 were classified as resilient (Figure S1). We further created other composite scores (avoidance-like score, social memory score, and PTSD-like score) related to other crucial features of PTSD, by z-normalizing data from different behavioral tests (open field (O.F), elevated place maze (E.P.M), 5-trials social memory (SM)) that mice underwent post-segregation, respectively, at days 31, 32, and 35. The described protocol provided 25–35% of susceptible mice and 65–75% of resilient mice. This protocol can be defined as safe and could be conceivably used both in rats and mice [91,94,95,96].

### 4.3. Selection of miRNAs

We retrieved the miRNAs targeting FKBP5 and BDNF by interpolating the predicted and validated human and murine data from miRecords (http://miRecords.umn.edu/miRecords, accessed on 10 October 2020), miRTarbase (http://miRTarBase.cuhk.edu.cn/, accessed on 10 October 2020) and ENCORI tools (http://starbase.sysu.edu.cn/index.php, accessed on 10 October 2020). Finally, the miRNA list was further filtered by a manually curated search on PubMed, taking into account the dysregulation of these miRNAs in neuropsychiatric phenotypes.

### 4.4. Tissue Collection

The day after the last behavioral experiment (36 days post-stress), all forty-six mice were sacrificed via cervical dislocation. We used thirty mice to dissect hippocampus and mPFC, and sixteen to dissect hypothalamus according to previously described protocols [97,98].

### 4.5. Total RNA Extraction

Total RNA was isolated from HIP, HT, and mPFC by using 1 mL Trizol Reagent/50–100 mg of tissue (Invitrogen Life Technologies, Waltham, MA, USA) following the manufacturer’s instructions. Briefly, after homogenization, 5 µg glycogen were added to increase RNA yield. Total RNA concentration and purity for each sample were evaluated by GeneQuant pro spectrophotometer (Biochrom, Cambridge, UK). RNA was stored at −80 °C until cDNA synthesis.

### 4.6. cDNA Synthesis and RT-qPCR

Single-stranded cDNA was synthesized from total RNA according to the manufacturer’s instructions by using the SuperScript III Reverse Transcriptase kit (Thermo Fisher Scientific, Waltham, MA, USA). We investigated mRNA expression in brain tissues through RT-qPCR by using iTaq Universal SYBR Green Supermix (Bio-Rad, Hercules, CA, USA), according to the manufacturer’s instructions. All RT-qPCR reactions were performed on a 7900HT Fast Real Time PCR System (Applied Biosystems, Life Technologies, Carlsbad, CA, USA). cDNA expression was evaluated through SDS RQ Manager 1.2 software (Applied Biosystems); normalization was performed using GAPDH as reference gene. No template samples were used as negative controls. Relative quantity (RQ) for each transcript was calculated according to the 2^−∆∆Ct^ method [99]. Technical triplicates were performed for each reaction. All RT-PCR reaction were performed in accordance with MIQE guidelines. PCR primers were designed by using the online tool Primer-BLAST (http://www.ncbi.nlm.nih.gov/tools/primer-blast/, accessed on 10 October 2020). Primer sequences are shown in Table 8.

### 4.7. TaqMan microRNA Assays

Specific single assays were performed for each selected miRNA, exploiting the miRNA-specific reverse transcription (TaqMan microRNA Reverse Transcription Kit; Thermo Fisher Scientific, Waltham, MA, USA) and Real-Time PCR (TaqMan Universal Master Mix II, no UNG; Thermo Fisher Scientific) by using Single TaqMan Assays (Thermo Fischer Scientific), according to the manufacturer’s instructions. RT-qPCRs were performed on a 7900HT Fast Real Time PCR System (Applied Biosystems, Life Technologies). The fold changes were calculated by the 2^−∆∆Ct^ method by using U6 snRNA as endogenous control. No template samples were used as negative control. Technical triplicates were performed for each reaction. All RT-qPCR reactions were performed in accordance with MIQE guidelines.

### 4.8. Computational Identification of miRNA Regulators

To computationally analyze the potential regulators of miRNAs, namely TFs and lncRNAs, we screened the TransmiR v2.0 (http://www.cuilab.cn/transmir, accessed on 10 October 2020) and LncBase v2.0 (https://diana.e-ce.uth.gr/lncbasev3, accessed on 10 October 2020) databases. From TransmiR, we retrieved the murine TF-miRNA regulations derived from ChIP-seq evidence, while LncBase provided the predicted interactions between miRNAs and lncRNAs in mouse.

### 4.9. Gene Ontology and Pathway Enrichment Analyses

Gene ontology and pathway enrichment analyses were performed to computationally investigate the biological effects of miRNA deregulation. First, we retrieved experimentally validated miRNA targets (where available) from DIANA Tarbase v8 (http://www.microrna.gr/tarbase, accessed on 10 October /2020); otherwise, predicted targets from microT-CDS (http://www.microrna.gr/webServer, accessed on 10 October 2020) were used. The list of miRNA targets was then submitted to the Database for Annotation, Visualization, and Integrated Discovery (DAVID) online tool (https://david.ncifcrf.gov/, accessed on 10 October 2020), which allowed us to screen different databases (Gene Ontology, Biocarta, KEGG, Reactome). Statistical over-representation was calculated by using Fisher’s exact test, Benjamini and Hochberg FDR Correction, *p* ≤ 0.05. Pathways showing false discovery rate (FDR) < 0.05 were considered statistically significant.

### 4.10. Statistical Analysis

Statistical significance of different mRNA and miRNA expression between groups was analyzed by one-way analysis of variance (ANOVA), followed by Tukey’s post hoc test for multiple comparisons. To assess data distribution normality, the D’Agostino-Pearson omnibus normality test was carried out. Expression data (RQ) were shown as a box and whiskers plot (min to max). No animals or samples were excluded from the analysis. Pearson’s correlation coefficients were calculated to statistically evaluate the strength and direction of the linear relationship among miRNA and mRNA expression values (RQ) and behavioral scores. Expression correlations among miRNAs were also investigated in mouse and human datasets from different tissues retrieved from Gene Expression Omnibus (GEO) datasets (https://www.ncbi.nlm.nih.gov/gds, accessed on 10 October 2020). For all analyses described in this section, *p*-values were two-sided and alpha was set to 0.05. All statistical analyses were performed using GraphPad Prism v7.0 (GraphPad Software, San Diego, CA, USA). To investigate the synergic effects of miRNAs on mRNA expression, linear regression models were built by using SPSS 23. The same statistical approach was used to evaluate the combined effect of expression variation of miRNAs + mRNAs, miRNAs only, and mRNAs only on modulation of behavioral scores.

## 5. Conclusions

In conclusion, we identified in a new PTSD-like model, brain-area-specific alterations of four miRNAs, potentially regulating two PTSD critical genes, BDNF and FKBP5. MiRNA modulation may underlie the epigenetic mechanisms defining resilience or susceptibility to stress by controlling the expression of BDNF and FKBP5 or other stress-related genes.

## Figures and Tables

**Table 1:** Expression correlations among miRNAs and BDNF/FKBP5.

Brain Area	Target	miR-15a-5p	miR-511-5p	miR-497a-5p	let-7d-5p
HIP	BDNF	−0.09 (0.65)	−0.04 (0.82)	−0.04 (0.80)	−0.18 (0.36)
	FKBP5	**−0.40 (0.03)**	**−0.45 (0.02)**	**−0.43 (0.02)**	−0.07 (0.69)
HT	BDNF	**0.66 (0.005)**	**0.73 (0.01)**	**0.53 (0.03)**	**0.62 (0.01)**
	FKBP5	**−0.69 (0.002)**	**−0.60 (0.01)**	**−0.62 (0.022)**	−0.38 (0.20)
mPFC	BDNF	−0.10 (0.60)	−0.38 (0.10)	−0.01 (0.94)	−0.32 (0.09)
	FKBP5	0.03 (0.85)	−0.20 (0.29)	0.06 (0.76)	−0.16 (0.39)

Pearson’s coefficient (r) and *p*-values (between brackets) are reported. Bold typed values are statistically significant. HIP: Hippocampus; HT: Hypothalamus; mPFC: medial Prefrontal Cortex.

**Table 2:** Linear regression models built on combinations of 4, 3, or 2 PTSD-related miRNAs.

Brain Area	Target	miRNA Combination	R	R^2^	*p*-Value
HIP	FKBP5	let-7d-5p, miR-15a-5p, miR-497a-5p, miR-511-5p	**0.657**	**0.431**	**0.024**
		miR-15a-5p, miR-497a-5p, miR-511-5p	**0.652**	**0.425**	**0.01**
		miR-497a-5p, miR-511-5p	**0.641**	**0.411**	**0.004**
	BDNF	let-7d-5p, miR-15a-5p, miR-497a-5p, miR-511-5p	0.191	0.037	0.936
		let-7d-5p, miR-15a-5p, miR-511-5p	0.191	0.037	0.841
		let-7d-5p, miR-15a-5p	0.186	0.035	0.656
HT	FKBP5	let-7d-5p, miR-15a-5p, miR-497a-5p, miR-511-5p	**0.749**	**0.56**	**0.044**
		miR-15a-5p, miR-497a-5p, miR-511-5p	**0.741**	**0.549**	**0.019**
		miR-15a-5p, miR-511-5p	**0.719**	**0.517**	**0.009**
mPFC	FKBP5	let-7d-5p, miR-15a-5p, miR-497a-5p, miR-511-5p	0.429	0.184	0.322
		let-7d-5p, miR-15a-5p, miR-511-5p	0.42	0.177	0.206
		let-7d-5p, miR-15a-5p	0.323	0.105	0.251
	BDNF	let-7d-5p, miR-15a-5p, miR-497a-5p, miR-511-5p	**0.597**	**0.357**	**0.038**
		let-7d-5p, miR-15a-5p, miR-511-5p	**0.587**	**0.345**	**0.019**
		let-7d-5p, miR-511-5p	**0.487**	**0.237**	**0.039**

For each combination of miRNAs, are reported: R-value, representing the coefficient of correlation; the R^2^ value, representing the proportion of variance of a dependent variable that may be explained by an (or more) independent variable(s); and the *p*-value. Bold typed values are statistically significant.

**Table 3:** Correlation matrix of miRNA expression in the different brain areas.

miRNAs	HIP			HT			mPFC		
	miR-15a-5p	let-7d-5p	miR-511-5p	miR-15a-5p	let-7d-5p	miR-511-5p	miR-15a-5p	let-7d-5p	miR-511-5p
let-7d-5p	**0.41 (0.03)**			**0.55 (0.02)**			**0.46 (0.01)**		
miR-511-5p	**0.38 (0.04)**	**0.39 (0.04)**		**0.70 (0.002)**	**0.63 (0.008)**		0.32 (0.09)	0.18 (0.36)	
miR-497a-5p	0.25 (0.19)	0.35 (0.06)	**0.64 (0.0003)**	0.39 (0.12)	0.38 (0.13)	**0.62 (0.01)**	**0.57 (0.001)**	**0.54 (0.003)**	0.14 (0.46)

Pearson’s coefficient (r) and *p*-values (between brackets) are reported. Bold typed values are statistically significant.

**Table 4:** Comparison matrix of TFs shared by miRNAs based on ChIp data.

miRNAs	miR-15a	let-7d	miR-497a
let-7d	**Cebpa, Spi1**		
miR-497a	Bcl11b, Brd4, Cbfb, **Cebpb**, Ctcf, Ep300, Erg, Ets1, Hdac2, Ikzf1, Max, Med1, Myc, Nelfe, Nr1d1, **Nr3c1**, Otx2, Pou5f1, Pparg, Rad21, **Rela**, Smarca4, **Spi1**, Suz12, Tfap4, Zfp281, Zfp384	**Spi1**	
miR-511	Batf, Btaf1, **Cebpa**, **Cebpb**, Mafb, **Nr3c1**, **Rela**, **Spi1**, Tcf12	**Cebpa, Spi1**	**Cebpb**, Fli1, **Nr3c1**, **Rela**, **Spi1**, Stat5a

TFs typed in bold are shared by several miRNA pairs.

**Table 5:** Comparison matrix of lncRNAs predicted to bind PTSD-related miRNAs.

	miR-15a-5p	let-7d-5p	miR-497a-5p
let-7d-5p	**Gm20605**, **Kcnq1ot1**, **Ptprv**, **Gm4117**		
miR-497a-5p	**Gm20605**, **Ptprv**, **ENSMUSG00000093535**, **Gm11579**, **Rab10os**, **Kcnq1ot1**, **Gm4117**	**Gm20605**, **Kcnq1ot1**, **Ptprv**, **Gm4117**, Mecomos	
miR-511-5p	**Kcnq1ot1**, **ENSMUSG00000093535**, **Gm4117**, **Gm11579**, ENSMUSG00000085287, **Rab10os**	**Kcnq1ot1**, Gm26856, **Gm4117**, Zfp950	**Kcnq1ot1**, **ENSMUSG00000093535**, **Gm4117**, **Gm11579**, **Rab10os**

LncRNAs typed in bold and underlined are shared by all miRNA pairs; lncRNAs typed in bold are shared by several miRNA pairs.

**Table 6:** Correlations between RNA expression and behavioral scores.

miRNAs/mRNAs	HIP			
	Arousal	Avoidance	Social Memory	PTSD-Like
miR-15a-5p	**−0.44 (0.02)**	−0.33 (0.08)	−0.12 (0.56)	−0.31 (0.11)
let-7d-5p	−0.08 (0.68)	0.26 (0.20)	0.30 (0.13)	0.16 (0.42)
miR-511-5p	**−0.44 (0.02)**	−0.14 (0.49)	−0.10 (0.61)	−0.34 (0.08)
miR-497a-5p	−0.22 (0.28)	−0.12 (0.56)	−0.10 (0.61)	−0.24 (0.22)
FKBP5	**0.39 (0.04)**	−0.04 (0.84)	−0.09 (0.65)	0.11 (0.61)
BDNF	**0.47 (0.01)**	−0.02 (0.89)	−0.10 (0.62)	0.27 (0.18)
	**HT**			
	**Arousal**	**Avoidance**	**Social Memory**	**PTSD-like**
miR-15a-5p	−0.26 (0.31)	−0.27 (0.30)	−0.11 (0.66)	−0.26 (0.31)
let-7d-5p	−0.12 (0.64)	−0.20 (0.45)	0.09 (0.72)	−0.07 (0.79)
miR-511-5p	−0.13 (0.62)	**−0.63 (0.007)**	−0.31 (0.23)	−0.39 (0.12)
miR-497a-5p	**−0.61 (0.01)**	**−0.57 (0.01)**	−0.37 (0.15)	**−0.67 (0.004)**
FKBP5	−0.02 (0.92)	0.22 (0.39)	0.27 (0.29)	−0.17 (0.51)
BDNF	−0.25 (0.33)	**−0.54 (0.02)**	−0.37 (0.15)	−0.46 (0.07)
	**mPFC**			
	**Arousal**	**Avoidance**	**Social Memory**	**PTSD-like**
miR-15a-5p	0.09 (0.65)	−0.10 (0.60)	0.10 (0.61)	0.04 (0.83)
let-7d-5p	0.11 (0.58)	−0.13 (0.53)	0.18 (0.38)	0.13 (0.52)
miR-511-5p	−0.09 (0.67)	−0.37 (0.07)	−0.09 (0.64)	−0.19 (0.34)
miR-497a-5p	−0.02 (0.92)	−0.27 (0.19)	−0.15 (0.45)	−0.15 (0.45)
FKBP5	0.27 (0.18)	0.16 (0.41)	0.18 (0.37)	0.30 (0.12)
BDNF	−0.27 (0.19)	0.16 (0.43)	0.28 (0.16)	0.02 (0.89)

Pearson’s coefficient (r) and *p*-values (between brackets) are reported. Bold typed values are statistically significant.

**Table 7:** Synergic effects of analyzed RNAs on behavioral scores.

Scores	miRNAs/mRNAs	HIPPOCAMPUS			HYPOTHALAMUS			mPFC		
		R	R^2^	*p*-value	R	R^2^	*p*-Value	R	R^2^	*p*-Value
Arousal score	miRNA + mRNAs	**0.72**	**0.519**	**0.032**	0.563	0.317	0.791	0.385	0.149	0.88
	MiRNA	0.442	0.196	0.335	**0.981**	**0.962**	**0.00022**	0.199	0.04	0.937
	mRNAs	**0.523**	**0.273**	**0.03**	0.274	0.075	0.761	0.103	0.011	0.885
Avoidance-like score	miRNA + mRNAs	0.529	0.28	0.402	0.728	0.53	0.449	0.503	0.253	0.632
	miRNA	0.349	0.122	0.606	0.711	0.506	0.304	0.488	0.238	0.247
	mRNAs	0.133	0.018	0.822	0.688	0.473	0.106	0.038	0.001	0.983
Social memory score	miRNA + mRNAs	0.691	0.477	0.058	0.884	0.782	0.094	0.541	0.293	0.526
	miRNA	0.512	0.263	0.172	**0.884**	**0.781**	**0.035**	0.207	0.043	0.928
	mRNAs	0.241	0.058	0.518	0.725	0.525	0.074	0.254	0.065	0.464
PTSD-like score	miRNA + mRNAs	0.699	0.488	0.05	0.69	0.476	0.541	0.531	0.282	0.554
	miRNA	0.502	0.252	0.194	**0.915**	**0.838**	**0.015**	0.33	0.109	0.681
	MRNAs	0.347	0.121	0.244	0.541	0.293	0.298	0.162	0.026	0.737

For each model, built on miRNAs+mRNA, only miRNAs, or only mRNAs, the R-value, representing the coefficient of correlation; the R^2^ value, representing the proportion of variance of a dependent variable that is/may be explained by an (or more) independent variable(s); and the *p*-value are reported. Bold typed values are statistically significant.

**Table 8:** PCR primer sequences of mRNAs analyzed in this study.

Gene	Forward Primer	Reverse Primer
FKBP5	5′-TGAGGGCACCAGTAACAATGG-3′	5′-CAACATCCCTTTGTAGTGGACAT-3′
BDNF	5′-GTTCGAGAGGTCTGACGACG-3′	5′-AGTCCGCGTCCTTATGGTTT-3′
GAPDH	5′-AGGTCGGTGTGAACGGATTTG-3′	5′-TGTAGACCATGTAGTTGAGGTCA-3′

## Data Availability

The data that support the findings of this study are available from the corresponding author upon reasonable request.

## Supplementary Materials

The following are available online at https://www.mdpi.com/article/10.3390/ijms22105157/s1, Figure S1: Experimental procedure conceived to shape the AIS model, which identified susceptible and resilient subpopulations.

## Abbreviations

ASR, acoustic startle reactivity; AIS, arousal-based individual screening; BDNF, brain derived neurotropic factor; BLC, basolateral amygdala complex; C, control; Cebpa, CCAAT/enhancer binding protein alpha; Cebpb, CCAAT/enhancer binding protein beta; CUMS, chronic unpredictable mild stress; DAVID, Database for Annotation, Visualization, and Integrated Discovery; E.P.M., elevated place maze; FDR, false discovery rate; FKBP5, FK506 binding protein 5; GEO, Gene Expression Omnibus; GR, glucocorticoid receptor; HIP, hippocampus; HPA, hypothalamic–pituitary–adrenal axis; HT, hypothalamus; Kcnq1ot1, KCNQ1 overlapping transcript 1; KEGG, Kyoto Encyclopedia of Genes and Genomes; mPFC, medial prefrontal cortex; miRNAs, microRNAs; ncRNAs, non-coding RNAs; O.F., open field; PTSD, post-traumatic stress disorder; R, resilient; SAM, sympathetic–adrenal– medullary system; Sp1, specificity protein 1; S, susceptible; TFs, transcriptional factors; Trk, tropomyosin receptor kinase; 5-trials (SM), 5-trial social memory test.
