# Supplementary material for: Dysregulation of miR-15a-5p, miR-497a-5p and miR-511-5p Is Associated with Modulation of BDNF and FKBP5 in Brain Areas of PTSD-Related Susceptible and Resilient Mice
Source: Int J Mol Sci. 2021 May 13;22(10):5157. doi: 10.3390/ijms22105157 (PMC8153003; doi:10.3390/ijms22105157)
Supplement: Supplementary file 1 [file ijms-22-05157-s001.zip › ijms-1208533-supplementary.pdf]

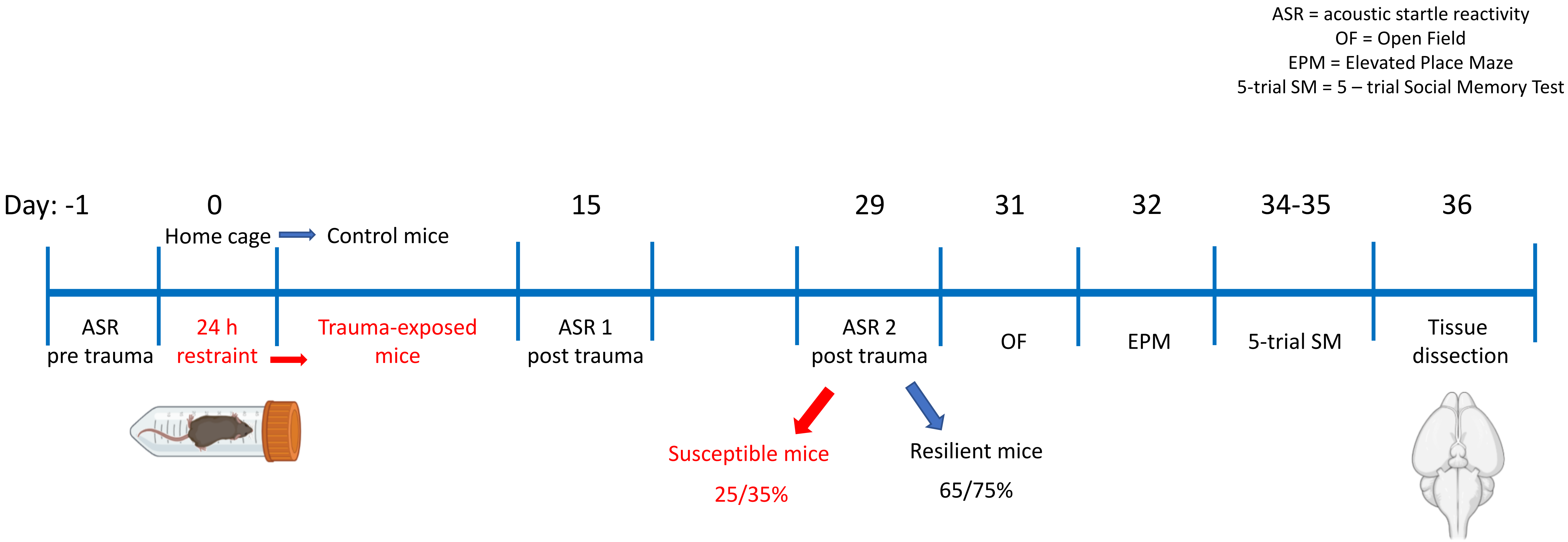

ASR = acoustic startle reactivity
OF = Open Field
EPM = Elevated Place Maze
5-trial SM = 5 – trial Social Memory Test
Day: -1
0
15
29
31
32
34-35
36
Home cage
Control mice
ASR
pre trauma
24 h
restraint
Trauma-exposed
mice
ASR 1
post trauma
ASR 2
post trauma
OF
EPM
5-trial SM
Tissue
dissection
Susceptible mice
25/35%
Resilient mice
65/75%
